# A Disproportionality Analysis for Association of Systemic Capillary Leak Syndrome with COVID-19 Vaccination Using the World Health Organization Pharmacovigilance Database

**DOI:** 10.3390/vaccines10060835

**Published:** 2022-05-25

**Authors:** Jin Park, Dongyeop Kim, Tae-Jin Song

**Affiliations:** Department of Neurology, Seoul Hospital, Ewha Womans University College of Medicine, 260 Gonghang-daero, Gangseo-gu, Seoul 07804, Korea; parkjin@ewha.ac.kr (J.P.); hap2028@ewha.ac.kr (D.K.)

**Keywords:** SARS-CoV-2, COVID-19 vaccination, adenoviral vector vaccine, mRNA-based vaccine, systemic capillary leak syndrome

## Abstract

Systemic capillary leak syndrome (SCLS) is a rare and potentially life-threatening disorder characterized by reversible plasma extravasation and vascular collapse. This study aimed to investigate the association between different types of COVID-19 vaccine and SCLS in a real-world setting. We used individual case safety reports of SCLS after COVID-19 vaccination from the WHO pharmacovigilance database, VigiBase. A disproportionality analysis of ChAdOx1 nCoV-19 and mRNA-based vaccines was performed. The information component (IC) and reporting odds ratio (ROR) were calculated from the entire database and viral vaccines data subset. A positive 95% lower end of the IC (IC_025_) value (>0) using Bayesian neural network analysis and lower end of the ROR 95% confidence interval (ROR_025_) ≥1 were defined as the ADR signal detection threshold. A total of 101 (0.004%) events of SCLS were identified. A significant potential signal of disproportionality of SCLS was noted in ChAdOx1 nCoV-19 when applied as the denominator for entire database (IC_025_ = 0.24, ROR_025_ = 1.23) and all viral vaccines (IC_025_ = 0.41, ROR_025_ = 1.59). No significant potential signal was noted for two mRNA-based vaccines as denominators for the entire database (IC_025_ = −0.49, ROR_025_ = 0.71) and all viral vaccines (IC_025_ = −0.32, ROR_025_ = 0.77). Contrary to ChAdOx1 nCoV-1, no safety signal for developing SCLS was identified for mRNA-based vaccines.

## 1. Introduction

SARS-CoV-2, including the delta and omicron variants, is still spreading around the world [1,2]. Herd immunity is important for epidemic control, and effective vaccination is needed to achieve herd immunity safely [3,4,5]. Several kinds of COVID-19 vaccinations have been developed, including mRNA-based vaccines and recombinant adenoviral vector vaccines, among which, a recombinant adenoviral vector vaccine (ChAdOx1 nCoV-19 [AstraZeneca, Cambridge, UK]) and two mRNA-based vaccines (BNT162b2 [Pfizer-BioNTech, Brooklyn, NY, USA] and mRNA-1273 [Moderna, Cambridge, MA, USA]) are still current and widely administered worldwide [6,7].

Systemic capillary leak syndrome (SCLS) is a rare disorder characterized by repeated extravasation or leakage of body fluids and proteins into the interstitial space [8]. Although no clear diagnostic criteria have been defined so far, the consensus is that SCLS can be strongly suspected when hypoalbuminemia, hemoconcentration, and hypotension are present [9]. Additionally, the pathophysiology of SCLS is not well known, and the prognosis was considered fair with about 70% of 5-year survival [10,11]. Upregulation of inflammatory and angiogenic molecules or a storm of an immune mechanism cascade related to overactivation of permeability in the vascular endothelium may be important mechanisms underlying SCLS development [9,10,12].

Diagnosis of idiopathic SCLS is based on the acute episodic attacks of systemic capillary hyperpermeability and by the presence (in >90% of cases) of monoclonal gammopathy [10], whereas secondary SCLS is caused by malignant hematological diseases, viral infections, and treatments such as chemotherapies and therapeutic growth factors [13]. It is important to differentiate between idiopathic and secondary SLCS because of different therapeutic implications [14]. While no specific drug has proven effective during the acute phase for both idiopathic and secondary SCLS [13], proper treatment for the underlying cause of secondary SCLS is important. On the contrary, in patients with idiopathic SCLS, the previous report showed the effect of the use of intravenous immunoglobulins as first-line prevention therapy [15].

Only a few case reports have discussed the potential association of COVID-19 vaccination with SCLS for either adenoviral vector vaccines or mRNA vaccines [16,17,18,19,20]. Since these case reports are mainly published for their unusual or interesting features, it is necessary to investigate associations between COVID-19 vaccination and SCLS in the real world. We hypothesized that COVID-19 vaccines would have a potential safety signal for SCLS. Here, we conducted a disproportionality analysis for a potential safety signal from BNT162b2, mRNA-1273, and ChAdOx1 nCoV-19 vaccines regarding SCLS using the World Health Organization’s (WHO) global pharmacovigilance database of individual case safety reports, VigiBase.

## 2. Methods

### 2.1. Study Design and Data Sources

Disproportionality analysis of adverse drug reactions (ADR) with BNT162b2, mRNA-1273, and ChAdOx1 nCoV-19 vaccines was performed using individual case safety reports from VigiBase, the WHO’s global deduplicated database, which includes reports from more than 130 countries [21,22]. VigiBase is managed by the Uppsala Monitoring Center (UMC) and collects information on suspected medication-related ADRs from national pharmacovigilance centers in each contributing country since 1967. Data come from many sources, including physicians, other healthcare professionals, pharmaceutical companies, patients, and post-marketing surveillance. This study of anonymized, prospectively updated, electronic data was approved by our institutional review board (EUMC-2021-08-021).

### 2.2. Procedures

In our observational case–control study, we extracted all cases of SCLS associated with BNT162b2, mRNA-1273, and ChAdOx1 nCoV-19 vaccines reported in VigiBase using the Medical Dictionary for Drug Regulatory Activities (MedDRA) preferred term (PT) on 1 February 2022 [23]. We defined SCLS based on the MedDRA PT. We obtained information on age group, sex, the kind of vaccine received, time to onset of reported SCLS, reporting region, severity, and outcomes.

### 2.3. Disproportionality Analysis

Disproportionality is evaluated by calculating the information component (IC) or reporting odds ratio using an entire database or an entire drug class according to each drug as a comparator. Detailed methods for calculating IC are provided in previous studies [24,25]. Because the entire database or entire drug classes according to each drug must be defined as the denominator to obtain IC, data for a direct drug-to-drug or vaccine-to-vaccine comparison cannot be acquired from the VigiBase [24,25]. Moreover, VigiBase from UMC makes no recommendations for drug–drug comparisons and states that there are no options for this type of comparison. Individual case safety reports from VigiBase were used for comparison with all viral vaccines. IC calculation was performed using a Bayesian confidence propagation neural network developed and validated by UMC [21]. In this way, ADR signals from a specific drug can be detected by comparing the possibility that there is a difference in the associated expected and observed drug ADRs based on the entire database or entire drug class according to each drug. IC_025_ is the 95% lower end of the IC. A positive IC_025_ value (>0) is the threshold for a significant signal detection as defined by the UMC [26]. For the sensitivity analysis, we also estimated the reporting odds ratio (ROR), which was frequently utilized for a potential safety signal before the concept of IC was established [27]. The lower end of a 95% confidence interval for the ROR (ROR_025_) ≥1 from the entire database or an entire drug class according to each drug as a control was defined as the threshold of ADR signal detection [28].

### 2.4. Statistical Analyses

Statistical analyses were executed using R software, version 3.3.3 (R Foundation for Statistical Computing, Vienna, Austria), and SAS, 9.4 version (SAS Inc., Cary, NC, USA). As recommended by VigiBase, BNT162b2 and mRNA-1273 vaccines were analyzed in combination to reduce selection bias. Categorical variables were presented as frequencies (%), and quantitative variables were presented as medians (interquartile range). Time to onset and outcome of SCLS were compared between vaccine types using the Kruskal–Wallis test with a subsequent Mann–Whitney U test. A *p*-value < 0.05/3 was set as a threshold for Bonferroni correction to correct for multiple-comparison bias. Subgroup analysis was performed with all viral vaccines (search terms in MedDRA and Vigiaccess ATC code: J07B) as the denominator.

## 3. Results

On 1 February 2022, the total number of ADR case reports in VigiBase was 2,8781,258 for all vaccines, and a total of 2,426,957 COVID-19 vaccine ADR reports (1,752,760 cases of mRNA-based vaccines [BNT162b2, mRNA-1273] and 674,197 ChAdOx1 nCoV-19) were identified. Among the overall ADR reports for COVID-19 vaccines, we identified 101 cases (0.004%) of SCLS. Among these ADR-reported cases for SCLS, 48 were from BNT162b2, 12 from mRNA-1273, and 41 from ChAdOx1 nCoV-19. The characteristics of each COVID-19 vaccine-associated SCLS are summarized in Table 1.

**Figure 1:**
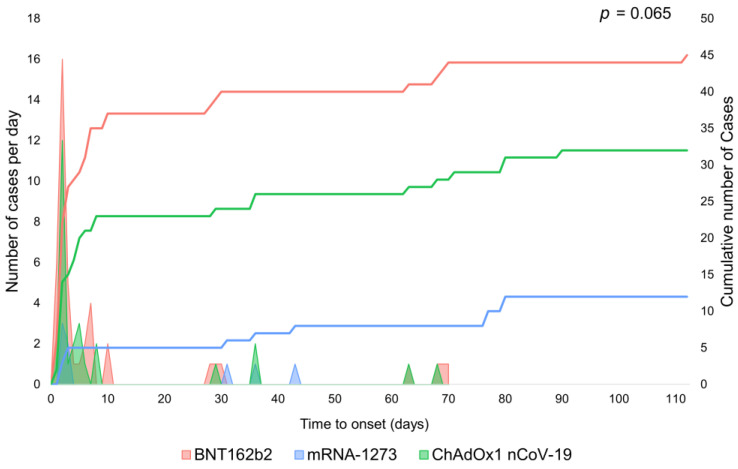
Time to onset of systemic capillary leak syndrome after vaccination with different types of COVID-19 vaccine. There was no significant difference observed among the vaccines.

SCLS as an ADR from ChAdOx1 nCoV-19 and mRNA-1273 was reported most among patients in the 45–64-year-old age group. For the BNT162b2 vaccine, it was most reported in the 18–44-year-old age group, closely followed by the 45–64-year-old age group. The region with the most reports of SCLS related to the ChAdOx1 nCoV-19 vaccine was Europe, whereas the region with the most reports of SCLS related to the BNT162b2 or mRNA-1273 vaccines was America. The median (interquartile range) time to onset of SCLS was 4 (2–31) days for ChAdOx1 nCoV-19, 3 (2–7) days for BNT162b2, and 34 (3–77) days for mRNA-1273 (*p* = 0.065 by Kruskal–Wallis test (Figure 1); post-hoc analysis of ChAdOx1 nCoV-19 versus BNT162b2, *p* = 0.4542 and ChAdOx1 nCoV-19 versus mRNA-1273, *p* = 0.1947 (Supplementary Figure S1)). Subgroup analysis regarding onset within 2 weeks showed no significant difference in time to onset between vaccines (Supplementary Figure S2).

**Figure 2:**
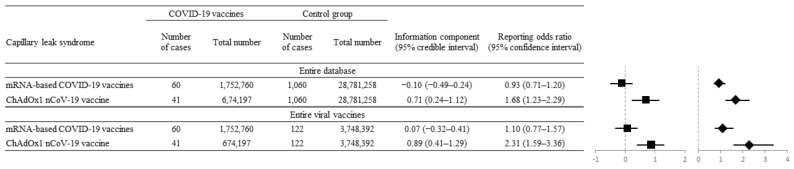
Disproportionality analysis between mRNA-based vaccines and ChAdOx1 nCoV-19 vaccine to compare systemic capillary leak syndrome (SCLS) occurrence. Forest plot with the reporting odds ratio (ROR, diamonds) and information component (IC, squares) values of mRNA-based (BNT162b2, mRNA-1273) and ChAdOx1 nCoV-19 vaccine-associated SCLS versus those from the entire database and all recorded viral vaccines. The ChAdOx1 nCoV-19 vaccine showed a significantly positive association with SCLS by IC_025_ of 0.71 (95% CI, 0.24–1.12) and ROR_025_ of 1.68 (95% CI, 1.23–2.29).

Considering IC_025_ and ROR_025_, a significant potential signal of disproportionality of SCLS was noted in ChAdOx1 nCoV-1 when applied as a denominator for the entire database (IC_025_ = 0.24, ROR_025_ = 1.23) and for all viral vaccines (IC_025_ = 0.41, ROR_025_ = 1.59). In contrast, no significant potential signal of disproportionality for SCLS was noted for two mRNA-based vaccines when applied as denominators for the entire database (IC_025_ = −0.49, ROR_025_ = 0.71) and for all viral vaccines (IC_025_ = −0.32, ROR_025_ = 0.77 (Figure 2)).

**Figure 3:**
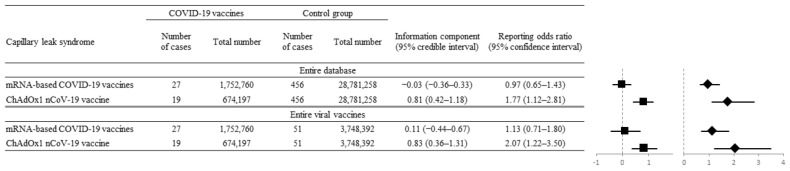
Disproportionality analysis using data reported by physicians and other healthcare professionals only. Forest plot with the reporting odds ratio (ROR, diamonds) and information component (IC, squares) values of mRNA-based (BNT162b2, mRNA-1273) and ChAdOx1 nCoV-19 vaccine-associated SCLS versus those from the entire database and all recorded viral vaccines. The ChAdOx1 nCoV-19 vaccine showed a significantly positive association with SCLS by IC_025_ of 0.81 (95% CI, 0.42–1.18) and ROR_025_ of 1.77 (95% CI, 1.12–2.81).

Additional disproportionality analysis was performed regarding data reported by physicians and other healthcare professionals only for minimizing reporting bias. It showed consistent results with the previous analysis using all kinds of reporting sources. A significant potential signal of disproportionality of SCLS was also noted in ChAdOx1 nCoV-1 when applied as a denominator for the entire database (IC_025_ = 0.42, ROR_025_ = 1.12) and for all viral vaccines (IC_025_ = 0.36, ROR_025_ = 1.22). On the contrary, no significant potential signal of disproportionality for SCLS was noted for two mRNA-based vaccines when applied as denominators for the entire database (IC_025_ = −0.36, ROR_025_ = 0.65) and for all viral vaccines (IC_025_ = −0.44, ROR_025_ = 0.71 (Figure 3)).

## 4. Discussion

The key findings of our study are that the ChAdOx1 nCoV-19 vaccine has a potential safety signal, whereas mRNA-based vaccines have no significant potential safety signal for the SCLS compared with the entire database and all viral vaccines according to real-world data from the WHO VigiBase that includes reporting from 130 countries.

SCLS is an extremely rare disease and is possibly underdiagnosed because of a lack of recognition and high mortality without treatment [9]. It is difficult to estimate the incidence of SCLS due to a dearth of large studies and lack of stringent diagnostic criteria [29]. A previous study reported 260 cases of idiopathic SCLS between 1960 and 2016 [9]. Secondary SCLS following vaccination was even less frequently reported before the era of COVID-19, with only one case report in a peritoneal dialysis patient [30], wherein the authors determined that the patient had two episodes of systemic SCLS in which mild symptoms developed 1 week after receiving the first influenza vaccine and more severe symptoms developed 5 weeks after receiving the second vaccine. Since the initial rollouts of COVID-19 vaccines in 2020, more than 10 billion doses have been administered globally. Several reports discussed the occurrence of SCLS within 4 days, commonly 1–2 days, after receiving the adenoviral vector or mRNA-based vaccines [16,17,18,19,20]. In the VigiBase dataset, most cases occurred within 1 week after vaccination. However, some safety reports were registered after 30 days and more after vaccination. This difference in time to onset from the existing case reports is difficult to explain because of the small number of case reports. Perhaps this is because case reports are mainly written for special cases, and the time to onset supplied by the VigiBase may not reflect the time of symptom onset but reporting day long after the diagnosis. In addition, since it is not possible to distinguish between the flare of idiopathic SCLS and secondary SCLS to vaccination in the database, different onset times may reflect the different classification of SCLS. Further study is needed regarding time to SCLS onset after COVID-19 vaccination.

Fifteen cases were identified as having SCLS after COVID-19 vaccination in the literature review; seven from ChAdOx1 nCoV-19, five from Ad26.COV2.S, two from BNT162b2, and one from mRNA-1273 [16,17,18,19,20]. Five of them (30%) who received an adenoviral vector vaccine subsequently died. Although they were heterogeneous, some with a history suggestive of SCLS or monoclonal gammopathy and some without SCLS-associated history, the estimated mortality was similar to or slightly higher than previous reports. In terms of mortality from idiopathic SCLS, it ranged from 20% to 30%. Dhir and colleagues estimated the current 5-year survival rate to be 70% [11]. Chambrun et al. reported that intravenous immunoglobulins improve the survival of patients with idiopathic SCLS, and overall 5- and 10-year survival rates from the European Clarkson Disease (EurêClark) registry were 78% and 69%, respectively [15].

Although a direct comparison between ChAdOx1 nCoV-19 vaccine and the mRNA-based COVID-19 vaccines was not conducted in this study, SCLS tended to be associated with the ChAdOx1 nCoV-19 vaccine, but not the mRNA-based COVID-19 vaccines. Previous case reports showed that SCLS developed after vaccination with mRNA-based COVID-19 vaccines [16,17,20]. Although we found that SCLS occurred in association with mRNA-based COVID-19 vaccines, the association was not significantly increased when compared with the entire database or all other viral vaccines.

The different potential safety signal of SCLS depending on the COVID-19 vaccine type is probably due to different mechanisms of action of vaccines. mRNA vaccines consist of mRNA that encodes the antigen of interest and is delivered into the cells via lipid nanoparticles, and this approach is safe because the mRNA carries a message but does not interact with the host genome [7]. No adjuvants or preservatives are used in mRNA vaccines [31]. The ChAdOx1 nCoV-19 vaccine uses a replication-incompetent modified chimpanzee DNA adenovirus as a vector that does not generate an immune response to the adenovirus itself [31]. Nevertheless, the possibility of the presence of pre-existing immunity against the adenovirus vector is the main disadvantage that could limit the effectiveness of these vaccines [7,32]. Thus, mRNA vaccines are considered to contain fewer particles that have a chance to be presented as antigens, and this may be associated with a less unexpected immune response. However, because our study cannot explain the mechanism of disease, a clear interpretation of the result is limited. Further research is needed on the mechanisms by which COVID-19 vaccines cause SCLS, particularly those associated with mRNA-based COVID-19 vaccines or adenovirus vector vaccines.

Our study has limitations that are mainly caused by methodological design. First, if the national drug monitoring center of each country does not report ADR, it cannot be confirmed by VigiBase. However, the merit of VigiBase is that rare ADR and generalized ADR information can be obtained from more than 130 countries. Second, the diagnosis of SCLS in this study may be inaccurate. VigiBase data are reported not only by physicians but also by other healthcare professionals or patients. There may have been a reporting bias according to different reporting sources, especially under-reporting because of a lack of recognition. Moreover, VigiBase does not validate laboratory findings such as hypoalbuminemia, hemoconcentration, and hypotension, which are needed to diagnose SCLS. Third, VigiBase data does not include clinical information about a history of suggestive of SCLS, underlying monoclonal gammopathy associated with idiopathic SCLS, or secondary causes other than vaccination. Thus, it is very hard to differentiate between flares of idiopathic SCLS and secondary SCLS. It also does not include the information on which dose of vaccination is associated with SCLS.

## 5. Conclusions

In conclusion, in contrast to the ChAdOx1 nCoV-1 vaccine, no potential safety signal for developing SCLS was noted in mRNA COVID-19 vaccines compared with the entire database. Moreover, the potential safety signal regarding SCLS may be similar between mRNA COVID-19 vaccines and other viral vaccines. It is important to recognize SCLS as a rare but potentially life-threatening disorder after the COVID-19 vaccination, possibly more associated with the ChAdOx1 nCoV-1 vaccine. More research is required for establishing diagnostic criteria for SCLS and elucidating the causal relationship between SCLS and the COVID-19 vaccines.

## Figures and Tables

**Table 1:** Demographics and characteristics of reported cases with systemic capillary leak syndrome (SCLS) according to type of COVID-19 vaccines.

Characteristics	Total (*n* = 101)	BNT162b2 (*n* = 48)	mRNA-1273 (*n* = 12)	ChAdOx1 nCoV-19 (*n* = 41)
Age, years				
≤11	0 (0)	0 (0)	0 (0)	0 (0)
12–17	1 (1)	1 (2)	0 (0)	0 (0)
18–44	13 (13)	9 (19)	1 (8)	3 (7)
45–64	36 (36)	9 (19)	11 (92)	16 (39)
65–74	19 (19)	7 (15)	0 (0)	12 (29)
≥75	7 (7)	3 (6)	0 (0)	4 (10)
Unknown	25 (25)	19 (40)	0 (0)	6 (15)
Sex				
Male	37 (37)	20 (42)	5 (42)	12 (29)
Female	60 (59)	28 (58)	7 (58)	25 (61)
Unknown	4 (4)	0 (0)	0 (0)	4 (10)
Location				
Africa	0 (0)	0 (0)	0 (0)	0 (0)
Americas	33 (33)	27 (56)	6 (50)	0 (0)
Asia	0 (0)	0 (0)	0 (0)	0 (0)
Europe	62 (61)	20 (42)	6 (50)	36 (88)
Oceania	6 (6)	1 (2)	0 (0)	5 (12)
Seriousness				
Yes	75 (74)	32 (67)	11 (92)	32 (78)
No	26 (26)	16 (33)	1 (8)	9 (22)
Time to onset (days)	3 [2–29]	3 [2–7]	34 [3–77]	4 [2–31]
Outcome				
Recovered	24 (24)	9 (19)	9 (75)	6 (15)
Recovered with sequelae	5 (5)	1 (2)	2 (17)	2 (5)
Recovering	19 (19)	11 (23)	0 (0)	8 (20)
Not recovered	17 (17)	5 (10)	1 (8)	11 (27)
Death	5 (5)	2 (4)	0 (0)	3 (7)
Unknown	31 (31)	20 (42)	0 (0)	11 (27)

Data are presented as number (%) or median [interquartile range]. Seriousness: resulting in significant disability/incapacity, requiring hospitalization, and fatality. Time to onset (days): the period from the date of vaccination to the reported onset of SCLS.

## Data Availability

The data will be available on request from the corresponding author.

## Supplementary Materials

The following supporting information can be downloaded at: https://www.mdpi.com/article/10.3390/vaccines10060835/s1. Figure S1: The time interval from vaccination to occurrence of systemic capillary leak syndrome for each vaccine type. Figure S2: Subgroup analysis representing the time interval within 2 weeks from vaccination to occurrence of systemic capillary leak syndrome for each vaccine type.
