# Supplementary material for: A Disproportionality Analysis for Association of Systemic Capillary Leak Syndrome with COVID-19 Vaccination Using the World Health Organization Pharmacovigilance Database
Source: Vaccines (Basel). 2022 May 25;10(6):835. doi: 10.3390/vaccines10060835 (PMC9227463; doi:10.3390/vaccines10060835)
Supplement: Supplementary file 1 [file vaccines-10-00835-s001.zip › vaccines-1703732-supplementary.pdf]

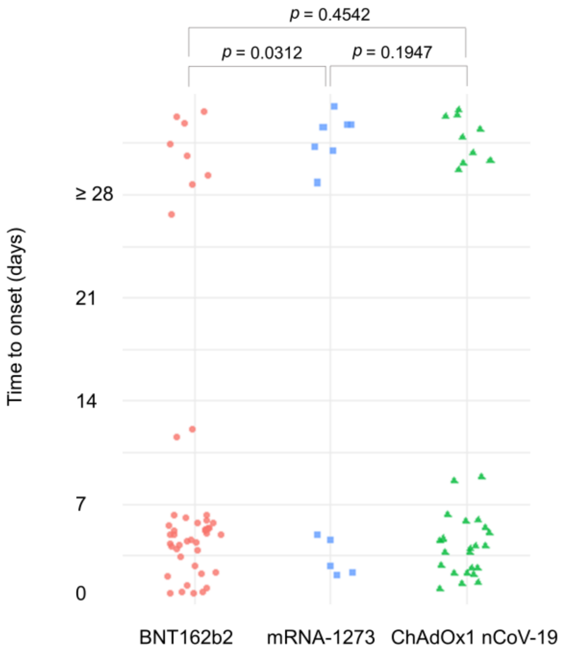

Figure S1. The time interval from vaccination to occurrence of systemic capillary leak syndrome for each vaccine type.

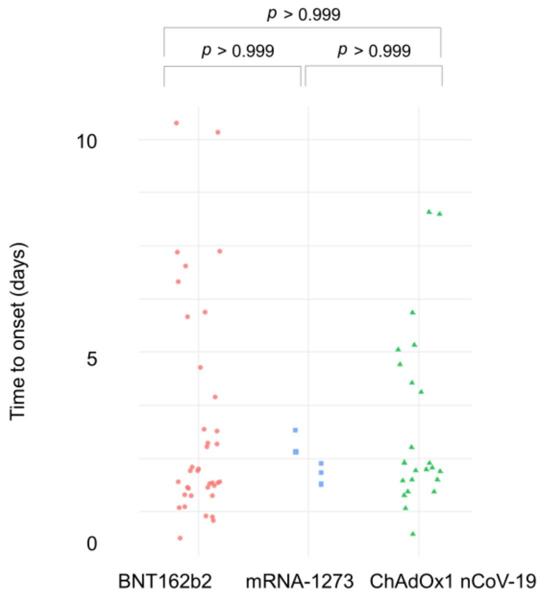

Figure S2. Subgroup analysis representing the time interval within 2 weeks from vaccination to occurrence of systemic capillary leak syndrome for each vaccine type.
